# QuEChERS-气相色谱-四极杆飞行时间质谱筛查大米中有机磷阻燃剂残留

**DOI:** 10.3724/SP.J.1123.2023.08022

**Published:** 2023-11-08

**Authors:** Kangcong LI, Jishuang YANG, Xiuqin LI, Yan GAO, Qinghe ZHANG

**Affiliations:** 1.中国计量科学研究院化学计量与分析科学研究所, 北京 100029; 1. Division of Chemical Metrology and Analytical Science, National Institute of Metrology, Beijing 100029, China; 2.中国信息通信研究院, 北京 100083; 2. China Academy of Information and Communications Technology, Beijing 100183, China

**Keywords:** QuEChERS, 气相色谱-四极杆飞行时间质谱, 有机磷阻燃剂, 筛查数据库, 大米, QuEChERS, gas chromatography-quadrupole time-of-flight mass spectrometry (GC-Q-TOF/MS), organophosphorus flame retardants (OPFRs), screening database, rice

## Abstract

基于QuEChERS-气相色谱-四极杆飞行时间质谱(GC-Q-TOF/MS)建立了大米中21种有机磷阻燃剂(OPFRs)的筛查方法,并对不同地区大米样品中的OPFRs残留情况进行分析。首先对21种OPFRs混合标准溶液(含量为0.1 μg/g)进行GC-Q-TOF/MS全扫描(扫描范围*m/z* 50~450),之后对21种OPFRs的裂解机理进行研究,获得化合物的保留时间、同位素丰度比、特征碎片分子式以及精确质量数等信息,建立OPFRs的标准谱库;采用QuEChERS方法对大米样品进行提取和净化,选择DB-5MS UI色谱柱,在优化的升温程序条件下,21种OPFRs在16 min内可完成色谱分离;在特征碎片离子数量≥2、精确质量窗口为±2×10^-5^(±20 ppm)、保留时间偏差为±0.2 min、离子丰度偏差小于20%的条件下,利用解卷积软件对样品全扫描数据进行分析,实现了大米样品中21种OPFRs的筛查。结果表明,对于烷基类和芳香类OPFRs,将加标水平从2 ng/g提升至10 ng/g时,检出率可分别提升22%和25%;在2 ng/g的加标水平下,氯化类OPFRs中仅磷酸三(2-氯异丙基)酯(TCIPP)未被检出,说明该类OPFRs即使在低浓度情况下也相对容易被确认。采用所建立的分析方法对不同地区的实际大米样品进行测定,共检出11种OPFRs,其中磷酸三甲酯(TMP)、磷酸三(异丁基)酯(TiBP)、磷酸三(3,5-二甲基苯基)酯(T35DMPP)的检出率最高,且不同地区大米样品中的OPFRs种类存在一定差异。本方法利用GC-Q-TOF/MS技术实现了大米样品中OPFRs的鉴定,为复杂基质样品中OPFRs的准确筛查提供了参考。

## Abstract (en)

Organophosphorus flame retardants (OPFRs) have emerged as good alternatives to brominated flame retardants, the use of which is globally restricted. In this study, a screening method based on QuEChERS-gas chromatography-quadrupole time-of-flight mass spectrometry (GC-Q-TOF/MS) was established for the determination of 21 OPFRs in rice. First, full scan (scanning range, *m/z* 50-450) was performed with a mixed standard solution of the 21 OPFRs (0.1 μg/g) by GC-Q-TOF/MS. The fragmentation pathways of these OPFRs were then investigated to explore their cleavage fragments, the interrelationships among fragments, and the possible cleavage modes of alkylated, chlorinated, and aromatic OPFRs. The retention times, isotopic abundance ratios, and molecular formulas of the characteristic fragments as well as the exact mass of the compounds were obtained to establish a mass spectral library of the OPFRs. Rice samples were extracted and purified by the QuEChERS method, and 0.5% formate acetonitrile solution was used as the extraction solvent; 4 g of magnesium sulfate, 1 g of sodium chloride, 0.5 g of disodium hydrogen citrate, and 1 g of sodium citrate as the extraction-salt combination; and 50 mg of primary secondary amine (PSA), 50 mg of octadecylsilane (C18), and 150 mg of magnesium sulfate as the purification materials. The chromatographic separation of the 21 OPFRs was completed within 16 min under optimized temperature program conditions on the DB-5MS UI column. The screening parameters were optimized, and a full scan of the samples was performed under the following conditions: number of characteristic fragment ions ≥2; accurate mass window=±2×10^-5^ (±20 ppm); retention time deviation=±0.2 min, and ion abundance deviation<20%. The developed method was applied to the screening 21 OPFRs in the samples. The results indicated that the matrix interference was greatly reduced by decreasing the extraction accurate mass window, thereby improving the signal-to-noise ratio of the analytes. The targets were extracted from the matrix interference and background noise using deconvolution software, which improved the match between the target compounds and the mass spectral library. The detection rates of alkyl and aromatic OPFRs increased by 22% and 25%, respectively, when the spiking level was increased from 2 to 10 ng/g. Among the chlorinated OPFRs, only tris(2-chloroisopropyl) phosphate (TCIPP) was not detected at a spiking level of 2 ng/g, indicating that chlorinated OPFRs could be identified even at low concentrations. The characteristic ions of the detected compounds matched those of the home-made mass spectral library well, indicating that the practical application of the home-made mass spectral library. The established screening method was applied in the determination of OPFRs in rice samples from different regions in China. A total of 11 OPFRs were detected, among which trimethyl phosphate (TMP), tri-iso-butyl phosphate (TiBP), and tris(3,5-dimethylphenyl) phosphate (T35DMPP) had the highest detection rates. These results indicate that these three OPFRs are widely used and can easily come into contact with rice samples through various routes. Differences in the types of OPFRs detected in the actual samples may be related to the types of OPFRs produced in local factories. OPFRs can be detected in rice samples by the developed GC-Q-TOF/MS screening method, which is helpful for the identification of OPFRs in complex matrix samples.

有机磷阻燃剂(organophosphorus flame retardants, OPFRs)对高分子材料具有阻燃和增塑作用,目前OPFRs作为传统溴系阻燃剂的替代品已被广泛应用^[1⇓-3]^。OPFRs通常以物理混合的工艺来参与生产加工,其极易通过磨损、浸出的方式释放到外界环境或附着在物体表面。近年来,OPFRs在水体^[4]^、灰尘^[5]^、淤泥^[6]^以及各种食品^[7⇓⇓-10]^中频繁检出,其分布遍及全球各个地区,不同地区受污染的程度和类型存在一定的差异,检出浓度覆盖多个数量级^[11]^。

大米是我国最主要的粮食作物,全国约有2/3的人口以大米为主食,但在食品中,大米受OPFRs的污染较为严重,可能会对人体健康造成影响^[12]^。大米样品相对复杂,在痕量分析大米样品中的OPFRs时,脂质、蛋白质和糖类等共提取成分会对测定造成干扰^[13]^,因此选择合适的前处理技术,有效去除大米样品中的杂质,对提取多组分OPFRs十分重要。OPFRs的样品前处理通常由提取和净化两个部分组成,常见的提取、净化方法包括加速溶剂萃取^[14]^、固相萃取^[15]^、分散固相萃取^[16]^、QuEChERS^[17]^和凝胶渗透色谱法^[18]^等。与传统的前处理技术相比,QuEChERS方法的分析速度更快,操作更加简便,有机溶剂消耗较少,无需加热且回收率高,近年来已广泛应用于食品中痕量有机物的前处理。合适的分离、检测方法可以降低化合物间的相互干扰和基质效应,从而提高OPFRs检测的灵敏度和选择性。以气相色谱(GC)或液相色谱(LC)与三重四极杆质谱、飞行时间质谱或静电场轨道阱质谱联用的检测方法在分析食品中OPFRs方面应用最广,如气相色谱-电子轰击源/化学电离源-串联质谱(GC-EI/CI-MS/MS)^[15]^、气相色谱-四极杆飞行时间质谱(GC-Q-TOF/MS)^[19]^、液相色谱-电喷雾电离源/大气压化学电离源-串联质谱(LC-ESI/APCI-MS/MS)^[7]^以及超高效液相色谱-高分辨质谱(UPLC-HRMS)^[20]^等;其中,GC-Q-TOF/MS具有高分辨率、高扫描速率、窄窗口数据提取及高效解卷积(deconvolution)识别等优势,能够获得大量的目标化合物信息^[21]^。Portolés等^[22]^使用GC-Q-TOF/MS技术,根据获得的碎片离子精确质量数来推断目标化合物特有的官能团和化学结构,基于测得的质谱图及所推断的特征碎片,对样品中的目标化合物进行筛查;该技术在全扫描模式下可测定目标化合物的精确质量数,通过建立特征离子精确质量数据库来降低检测过程中的假阳性率和假阴性率,从而提高定性分析的准确性,在食品污染物痕量多残留分析中表现出了突出的优势^[23]^。

OPFRs作为一种新兴的污染物,其检测技术目前还不够完善,借助质谱数据库对OPFRs进行筛查可有效提高检测效率和准确度。本文针对大米样品,利用QuEChERS前处理方法和GC-Q-TOF/MS检测技术的优势,建立了大米中21种OPFRs的筛查方法。实验使用电子轰击电离源,在全扫描模式下测定21种OPFRs,并建立了质谱特征离子精确质量数据库;同时对样品前处理方法、仪器条件和筛查参数进行了考察和优化,为准确筛查复杂基质中的OPFRs提供了技术手段。

## 1 实验部分

### 1.1 仪器、试剂与材料

7890B-7200气相色谱-四极杆飞行时间质谱联用仪(美国Agilent公司); Sartorius SE 2分析天平(德国Sartorius公司); MX-S涡旋仪(美国Scilogex公司); CR21GⅢ离心机(日本Hitachi公司); AutoEVA-20L氮吹仪(睿科集团股份有限公司)。

21种OPFRs标准品,分为3类,烷基类OPFRs:磷酸三甲酯(TMP)、磷酸三异丙酯(TiPP)、磷酸三丙酯(TnPP)、磷酸三(异丁基)酯(TiBP)、磷酸三正丁酯(TnBP)、磷酸三(3-丁氧基乙基)酯(TBOEP)、磷酸三戊酯(TPeP)、磷酸三(2-乙基己基)酯(TEHP);氯化类OPFRs:磷酸三(氯乙基)酯(TCEP)、磷酸三(2-氯异丙基)酯(TCIPP)、磷酸三(氯丙基)酯(TCPP)、磷酸三(1,3-二氯-2-丙基)酯(TDCIPP);芳香类OPFRs:二丁基苯基磷酸酯(dBPhP)、磷酸二苯酯(BdPhP)、磷酸三苯酯(TPHP)、磷酸(2-乙基己基)二苯酯(EHDPP)、磷酸邻三甲苯酯(*o*-TCP)、磷酸间三甲苯酯(*m*-TCP)、磷酸对三甲苯酯(*p*-TCP)、磷酸三(3,5-二甲基苯基)酯(T35DMPP)、磷酸甲苯基二苯酯(CDPP)(德国Dr. Ehrenstorfer公司、加拿大TRC公司、美国Chemservice公司、曼哈顿生物科技有限公司、德国安谱公司、美国Supelco公司、挪威Chiron公司、加拿大CDN Isotopes公司); 无水硫酸镁、氯化钠(分析纯,国药集团试剂有限公司);柠檬酸缓冲盐(分析纯,天津博纳艾杰尔公司);十八烷基硅烷(C18)、*N*-丙基乙二胺(PSA)、氧化锆涂层的二氧化硅(Z-Sep)(分析纯,美国Supelco公司)。大米样品收集自中国江西、广西和湖北。

### 1.2 标准溶液的配制

OPFRs标准储备溶液的配制:根据OPFRs的溶解性和极性选用甲醇或甲苯作为溶剂,分别配制100 μg/g的21种OPFRs标准储备溶液,置于4 ℃下保存。

21种OPFRs混合标准溶液的配制:选用乙酸乙酯作为溶剂,对21种OPFRs标准储备溶液进行混合、稀释,配制成1 μg/g的OPFRs混合标准溶液,再通过逐级稀释,分别配制0.5、1、10、20、50、100、200 ng/g的OPFRs系列混合标准溶液。

### 1.3 样品前处理

参照之前的研究工作^[24]^,样品前处理流程如下:(1)准确称量1 g匀质的大米样品置于50 mL离心管中,静置30 min; (2)向样品中加入10 mL 0.5%甲酸乙腈溶液,摇匀并在20 ℃水浴下超声处理5 min,再向离心管中加入4 g无水硫酸镁、1 g氯化钠、0.5 g柠檬酸氢二钠、1 g柠檬酸钠提取盐包,涡旋1 min,在10000 r/min下离心5 min,提取上清液;(3)将提取液转移至含有50 mg PSA、50 mg C18和150 mg无水硫酸镁的离心管中,涡旋1 min,在12000 r/min下离心5 min; (4)将离心后的上清液用聚四氟乙烯滤膜(0.22 μm)过滤,之后在温和的氮气条件下进行干燥,再用1 mL乙酸乙酯复溶,在14000 r/min下离心5 min,取0.8 mL上清液备用。最后取1 μL上清液样品注入GC-Q-TOF/MS系统,每批样品中均加入一份过程空白。

### 1.4 GC-Q-TOF/MS检测条件

色谱柱:Agilent DB-5MS UI石英毛细管柱(30 m×0.25 mm×0.25 μm);载气:氦气(99.999%);流速:1.0 mL/min;进样口温度:280 ℃;进样体积:1 μL,不分流进样。升温程序:在50 ℃保持1 min;以20 ℃/min升温至280 ℃,保持1 min;以30 ℃/min升温至300 ℃,保持10 min。

离子源:EI源;电离能:50 eV;灯丝电流:20 μA;四极杆温度:150 ℃;离子源温度:230 ℃;溶剂延迟:3.5 min;采用全扫描采集模式,采集速率为1 spectrum/s,使用Mass Hunter定性分析软件进行数据分析。

### 1.5 筛查参数

在特征碎片离子数量≥2、精确质量窗口为±2×10^-5^(±20 ppm)、保留时间偏差为±0.2 min、离子丰度偏差小于20%的条件下,利用解卷积软件对样品的全扫描数据进行分析。

### 1.6 OPFRs标准谱库的建立

21种OPFRs的混合标准溶液(含量为0.1 μg/g)经气相色谱分离后,进入四极杆飞行时间质谱,并在EI源电离下产生碎片离子。对电离后产生的所有碎片子离子进行全扫描,质量扫描范围为*m/z* 50~450。对OPFRs的裂解机理进行研究,获得各化合物的保留时间、同位素丰度比、特征碎片分子式以及精确质量数等信息,同时将所有OPFRs的名称、简称、分子式和CAS号等信息导入谱库编辑器,并用CSV格式输出保存,建立OPFRs的靶向快速筛查标准谱库。通过读取标准谱图中碎片离子的精确质量数,建立了特征离子的精确质量数据库,特征离子精确质量数据库是对样品中化合物进行筛查时的基础^[21]^。21种OPFRs的特征离子信息见表1。

**表1:** 21种OPFRs的特征离子信息

Compound	Molecular formula	CAS No.	t_R_/min	Fragment ions (m/z)		
				Ion 1	Ion 2	Ion 3
Trimethyl phosphate (TMP)	C_3_H_9_O_4_P	512-56-1	4.042	110.0111	80.0006	94.9883
Triethyl phosphate (TiPP)	C_9_H_21_O_4_P	513-02-0	6.144	98.9833	124.9990	141.0297
Tri-n-propyl phosphate (TnPP)	C_9_H_21_O_4_P	513-08-6	7.511	98.9834	141.0301	123.0197
Tri-iso-butyl phosphate (TiBP)	C_12_H_27_O_4_P	126-71-6	8.379	98.9834	223.0627	155.0458
Tri-n-butyl phosphate (TnBP)	C_12_H_27_O_4_P	126-73-8	9.196	98.9835	155.0458	211.1085
Tris(chloroethyl) phosphate (TCEP)	C_6_H_12_Cl_3_O_4_P	115-96-8	9.896	248.9840	250.9804	204.9571
Tris(2-chloroisopropyl) phosphate (TCIPP)	C_9_H_18_Cl_3_O_4_P	13674-84-5	10.063	124.9989	98.9830	201.0069
Dibutyl phenyl phosphate (dBPhP)	C_14_H_23_O_4_P	2528-36-1	10.513	175.0147	94.0405	187.0147
Tripentyl phosphate (TPeP)	C_15_H_33_O_4_P	2528-38-3	10.714	98.9833	169.0618	239.1396
Tris(chloropropyl) phosphate (TCPP)	C_9_H_18_Cl_3_O_4_P	1067-98-7	11.614	98.9840	139.0155	174.9922
Butyl diphenyl phosphate (BdPhP)	C_16_H_19_O_4_P	2752-95-6	11.789	251.0468	94.0413	170.0708
Tris(1,3-dichloro-2-propyl) phosphate (TDCIPP)	C_9_H_15_Cl_6_O_4_P	13674-87-8	12.648	98.9833	74.9989	190.9415
Tris(2-butoxyethyl) phosphate (TBOEP)	C_18_H_39_O_7_P	78-51-3	12.932	85.0644	124.9991	199.0725
Triphenyl phosphate (TPHP)	C_18_H_15_O_4_P	115-86-6	13.049	326.0677	325.0614	215.0246
2-Ethylhexyl-diphenyl phosphate (EHDPP)	C_20_H_27_O_4_P	1241-94-7	13.115	251.0452	250.0365	94.0399
Tris(2-ethylhexyl) phosphate (TEHP)	C_24_H_51_O_4_P	78-42-2	13.182	98.9831	113.1314	71.0851
Tri-o-tolyl-phosphate (o-TCP)	C_21_H_21_O_4_P	78-30-8	13.949	165.0689	368.1160	179.0849
Tri-m-tolyl-phosphate (m-TCP)	C_21_H_21_O_4_P	563-04-2	14.233	368.1158	367.1081	165.0691
Tri-p-tolyl-phosphate (p-TCP)	C_21_H_21_O_4_P	78-32-0	14.700	368.1157	367.1077	107.0481
Tris(3,5-dimethylphenyl) phosphate (T35DMPP)	C_24_H_27_O_4_P	25653-16-1	15.300	410.1647	395.1413	91.0538
Cresyl diphenyl phosphate (CDPP)	C_19_H_17_O_4_P	26444-49-5	15.676	340.0838	247.0503	229.0397

### 1.7 质量保证与质量控制

在OPFRs的检测过程中常伴随空白污染,因此对实验过程进行严格的质量控制和保证是十分必要的。对每批样品的样品空白、过程空白和溶剂空白进行分析,使用基质匹配标准溶液校正样品回收率,从而控制和保证检测结果的准确性。此外,样品均平行制备3份,每份样品平行测定3次。将全氟三丁胺(PFTBA)用于日常质谱校准,在保证仪器质量校准偏差不高于5 ppm的情况下对目标分析物进行测定,以确保分析物离子碎片质量的准确性。

## 2 结果与讨论

### 2.1 QuEChERS前处理方法的优化

前处理方法参照本研究组的相关工作^[24]^,研究结果表明,使用0.5%甲酸乙腈溶液作为提取溶剂可使回收率明显升高;选择带有缓冲盐的提取盐包可以维持体系pH的稳定;使用PSA+C18净化吸附剂可降低大米样品中糖类、脂肪和一些有机酸对实验产生的不利影响。因此,实验选择0.5%甲酸乙腈作为提取溶剂,使用4 g无水硫酸镁、1 g氯化钠、0.5 g柠檬酸氢二钠、1 g柠檬酸钠作为提取盐包,使用50 mg PSA和50 mg C18作为净化材料。

### 2.2 仪器条件的优化

#### 2.2.1 色谱柱的选择

为获得最佳分析物响应和较好的分离度,在其他色谱条件保持不变的情况下,分别比较了DB-5MS(30 m×0.25 mm×0.25 μm)与DB-5MS UI色谱柱对3类OPFRs峰面积响应值的影响。结果表明,使用DB-5MS UI色谱柱时,OPFRs有更高的响应,尤其对于烷基类OPFRs效果更为明显。这可能是由于DB-5MS UI色谱柱在高柱温条件下也能保持较低的柱流失,有效降低了检测过程中的干扰。

图1为分别使用DB-5MS和DB-5MS UI色谱柱检测相同浓度OPFRs时的色谱图。可以看出,DB-5MS UI色谱柱对TMP有较好的选择性和灵敏度,噪声信号也有所降低;同时,DB-5MS UI色谱柱对TEHP有较好的保留,弥补了使用DB-5MS色谱柱产生分叉峰的现象,峰形得到明显改善,TBOEP的信噪比也得到了提高。

**图1:**
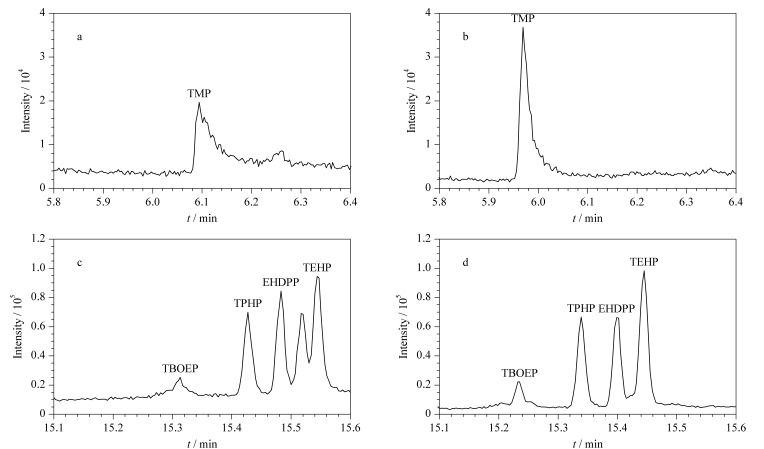
(a, c)DB-5MS和(b, d)DB-5MS UI色谱柱分离相同浓度OPFRs时的色谱图

#### 2.2.2 升温程序条件的优化

柱温是影响组分分离的重要因素,其对组分的峰形也有很大的影响。为了提高OPFRs各化合物间的分离度,常通过降低初始温度、改变升温速率或在某温度下保持一段时间等方法实现。对于沸点较低的TMP,其出峰时间较早,初始温度过高可能影响其在色谱柱上的保留效果;而对于沸点接近的EHDPP和TEHP,二者出峰时间相近,无法在色谱柱上有效分离;此外,对于沸点较高的OPFRs,则需要在最终温度下保持一段时间。

实验考察了两种升温程序条件,升温程序1:在60 ℃保持1 min,以20 ℃/min升温至300 ℃,保持10 min;升温程序2:在50 ℃保持1 min,以20 ℃/min升温至280 ℃,保持1 min,以30 ℃/min升温至300 ℃,保持10 min。在两种升温程序下比较了TMP、EHDPP、TEHP等化合物的峰形、分离度及灵敏度,图2为升温程序1和2条件下OPFRs的色谱图。可以看出,初始温度从60 ℃降低至50 ℃后,TMP的峰形得到了极大改善,响应强度也明显提高。在升温程序1的条件下,EHDPP和TEHP未能完全分离,其分离度仅为0.91;在升温程序2的条件下,4种化合物的分离度均达到1.26以上,并且EHDPP和TEHP的响应强度也有所提高。通过降低初始温度和增加高温条件下的保持时间,目标分析物无论在峰形还是灵敏度方面都得到了改善,且21种OPFRs在16 min内可完成色谱分离。因此,选取升温程序2对OPFRs进行分析测定。

**图2:**
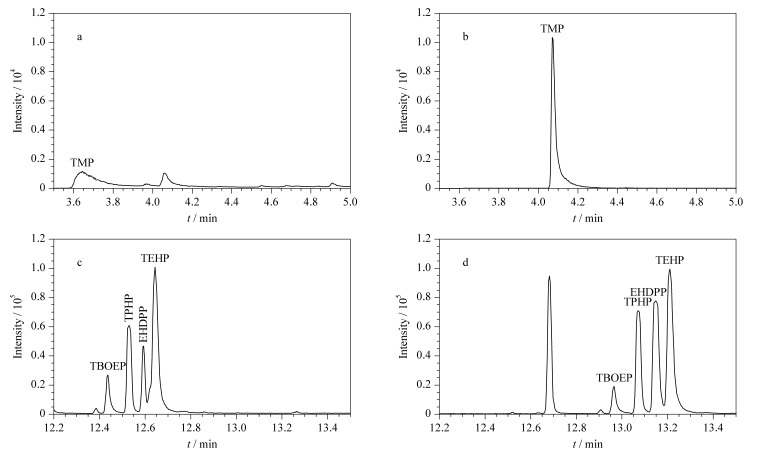
不同升温程序下OPFRs的色谱图

### 2.3 筛查参数的优化

使用标准谱库检索可以快速筛查和定性食品基质中的OPFRs。首先需要充分了解OPFRs的质谱行为,探究其裂解规律,建立OPFRs的标准质谱数据库。在对样品中的化合物进行鉴定时,通过设定保留时间偏差、精确质量窗口、同位素丰度偏差等,再经过解卷积和背景扣除处理,将所获得的质谱图与自建标准谱库进行比对分析,将谱库匹配度作为定性参考,从而降低假阳性或假阴性结果的产生。

#### 2.3.1 精确质量窗口

飞行时间质谱可通过减小精确质量窗口来降低基质干扰,从而提高分析物的信噪比,为复杂样品的痕量筛查提供了有利条件。在全扫描模式下,对大米基质匹配标准溶液中的21种OPFRs进行检测。在不同质量窗口下,对烷基类OPFRs特征碎片离子的理论质量数(*m/z* 98.9842)进行提取。如图3所示,以TiPP为例,在精确质量窗口分别为±500、±100、±20 ppm的条件下,TiPP(保留时间为8.485 min)的信噪比分别为1.6、55.7、218.7。在质量窗口为±500 ppm时,目标分析物的色谱峰包埋在噪声信号中而无法检出;当精确质量窗口降低至±100 ppm时,目标分析物附近的基线噪声干扰明显降低;当精确质量窗口降低至±20 ppm时,目标分析物的峰形得到了极大改善,灵敏度、选择性及辨析度也得到了有效提高。通过质量窗口提取氯化类和芳香类OPFRs时也得到了相同的结论。

**图 3:**
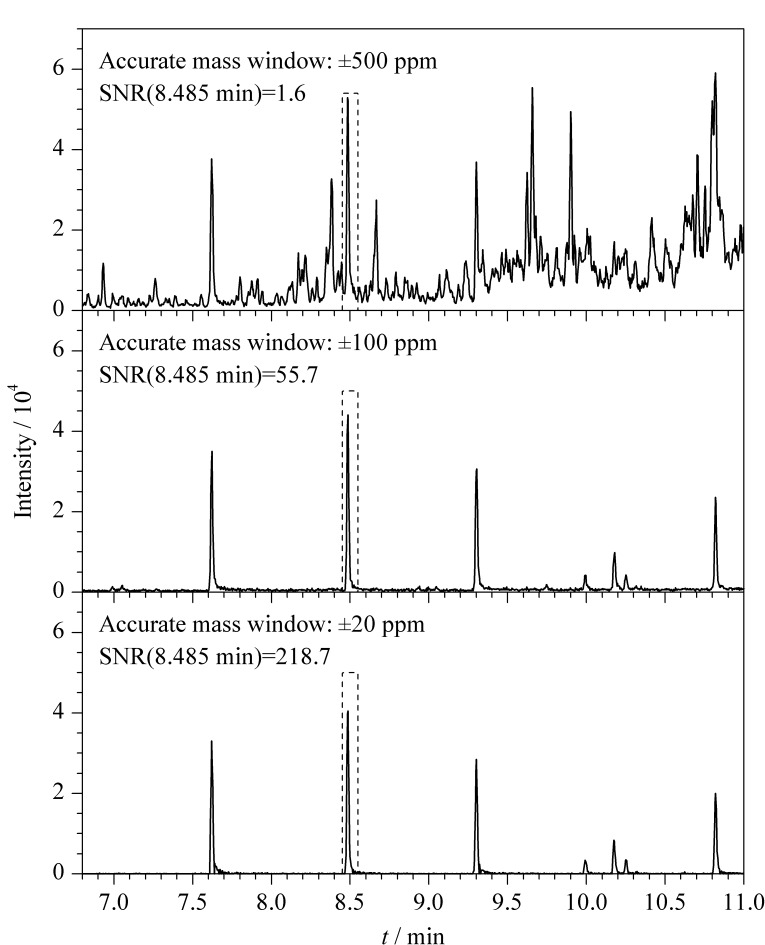
不同精确质量窗口下TiPP的色谱图

此外,实验还考察了精确质量窗口分别为±2、±5、±10、±15、±20 ppm时,几种典型OPFRs的响应曲线。从图4可以看出,质量窗口为±2~±10 ppm时,随着质量窗口的扩大,目标分析物的响应值逐渐增大,而当继续扩大质量窗口至±20 ppm时,响应值基本趋于平稳。为了保证目标分析物的灵敏度,使其不受基线噪声的干扰,并提高筛查匹配率,实验选择±20 ppm为最佳精确质量窗口。

**图 4:**
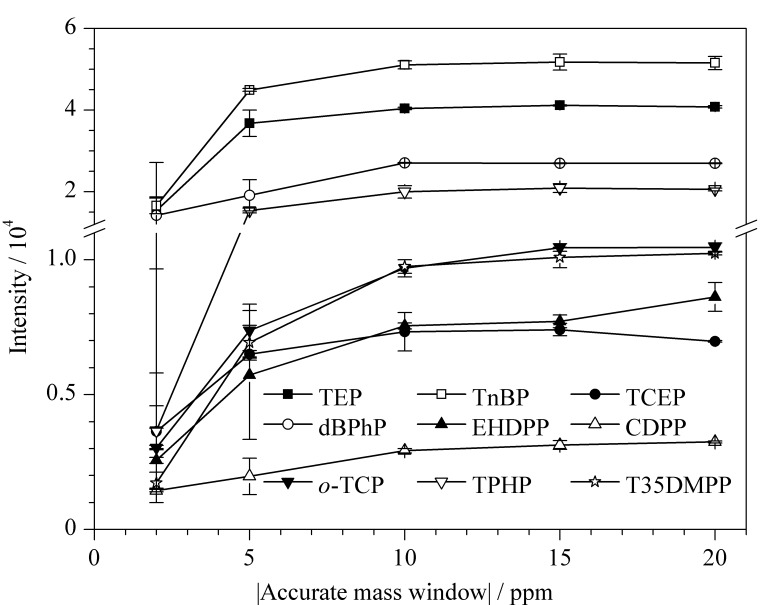
不同精确质量窗口下OPFRs的响应值(*n*=3)

#### 2.3.2 数据解卷积

由于样品中共提取物的存在以及样品在运行过程中产生的柱流失,在采用全扫描采集模式分析时,所得到的质谱图实际为包含了多种组分的组合图,这可能会导致筛查目标化合物时的匹配分值较低,甚至无法识别目标化合物。解卷积可以从紧密相邻的共洗脱峰中提取目标化合物的信号,提高目标化合物与质谱库的匹配分值,且有利于分析被基质掩盖的痕量组分^[19]^。

基于Mass Hunter定性分析软件,利用解卷积对大米样品中的OPFRs残留进行分析。以*o*-TCP为例,在全扫描模式下的大米样品提取液总离子流色谱图中,*o*-TCP的色谱峰被掩盖在基质干扰组分中,无法辨认;通过解卷积处理,可获得无背景干扰的*o*-TCP色谱峰。谱库检索结果显示,经过解卷积处理后的质谱图与自建标准谱库有较好的匹配,实现了对*o*-TCP的定性分析。

### 2.4 分析方法验证

#### 2.4.1 基质效应的评价

共洗脱成分在电离过程中干扰分析物,使分析物信号抑制或增强的现象称为基质效应(matrix effect, ME),基质效应会导致测量结果偏高或偏低。实验考察了21种OPFRs在大米样品中的基质效应,ME=*a*_1_/*a*_2_×100%,其中*a*_1_为OPFRs在基质匹配标准溶液中的响应强度,*a*_2_为OPFRs在空白溶剂标准溶液中的响应强度。当ME>100%时,表示存在基质增强效应;当ME<100%时,表示存在基质抑制效应;当80%<ME<120%时,说明基质的影响较小。实验结果表明,21种OPFRs的基质效应为97%~121%,大部分化合物受基质的影响较小,为筛查过程中的参数设置以及样品检测提供了有利条件。

#### 2.4.2 筛查条件的验证

根据欧盟对食品和饲料中农药残留的相关规定(SANTE 12682-2019^[25]^)以及我国农业部发布的《饲料中风险物质的筛查与确认导则》^[26]^等,在进行风险物质的筛查方法开发时,对于化合物的回收率不作严格要求。因此实验着重考察了OPFRs的筛查检出限及筛查参数,将检测到至少两种主要特征碎片离子、保留时间偏差为±0.2 min、精确质量窗口为±20 ppm、离子丰度偏差小于20%的结果判定为检出该OPFRs,符合该筛查参数的最小浓度为筛查检出限。例如,在大米样品中添加100 ng/g的TCPP,其4个特征离子的提取离子色谱图及谱库匹配质谱图如图5所示。从图5a可以看出,TCPP的4个特征离子均有检出,并且与特征离子理论精确质量数相比,质量精度偏差均小于5 ppm;从图5b可以看出,TCPP的特征碎片离子丰度与OPFRs谱库中的丰度基本一致。

**图 5:**
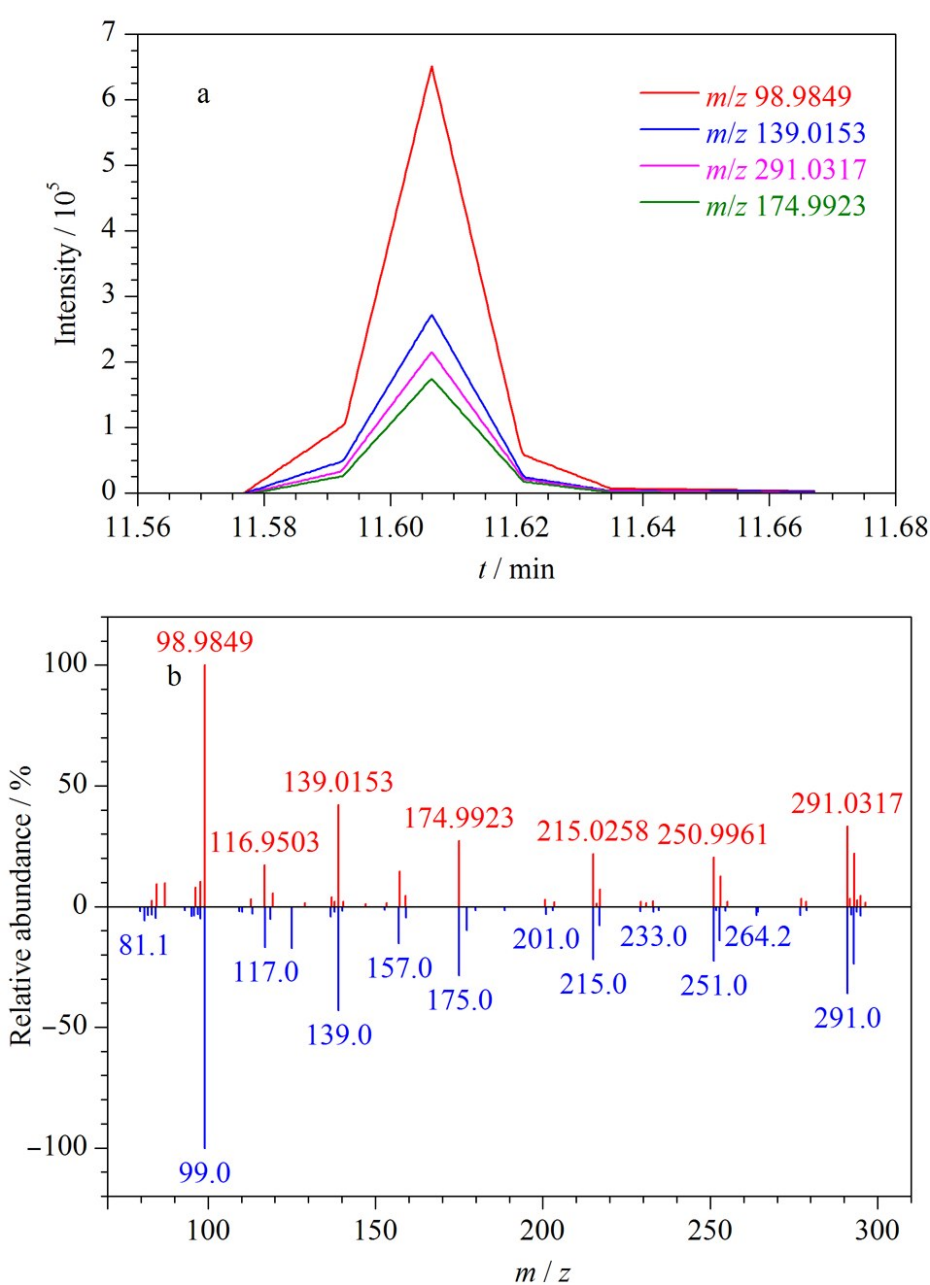
(a)TCPP特征碎片离子的色谱峰及其(b)与OPFRs标准质谱库的比对

按照上述方法,在大米样品中添加含量分别为2、10、100 ng/g的21种OPFRs混合标准溶液,并进行筛查。结果表明,在2 ng/g的添加水平下,筛查到5种烷基类OPFRs、3种氯化类OPFRs和4种芳香类OPFRs,未筛查到TiPP、TEHP、TnBP、TPeP、TCIPP、*p*-TCP、BdPhP、T35DMPP和CDPP;在10 ng/g的添加水平下,筛查到7种烷基类OPFRs、4种氯化类OPFRs和6种芳香类OPFRs,未筛查到TEHP、TPeP、T35DMPP和CDPP;而在100 ng/g的添加水平下,21种OPFRs全部被检出。OPFRs的添加水平越高,被筛查到的几率就越大,尤其对于烷基类和芳香类OPFRs,添加水平从2 ng/g提升至10 ng/g时,检出率分别提升了22%和25%。氯化类OPFRs在低加标水平下也相对容易被确认,在2 ng/g下仅TCIPP未被检出,这可能是因为该类化合物的碎片离子较丰富,目标物匹配值较高。

### 2.5 大米样品筛查

利用以上优化的筛查流程和参数,对样品的全扫描数据进行处理和分析后,在自建标准谱库中进行检索。在3个主要受OPFRs污染地区的大米样品中,共检出11种OPFRs。在江西大米样品中检出6种OPFRs,分别为TMP、TiBP、TnBP、TCEP、EHDPP、T35DMPP;在广西大米样品中检出4种OPFRs,分别为TMP、TiBP、TCIPP、T35DMPP;在湖北大米样品中检出8种OPFRs,分别为TMP、TiBP、TEHP、TCEP、TCPP、TDCIPP、dBPhP、T35DMPP;其中TMP、TiBP和T35DMPP在3个地区的大米样品中均有检出,说明这3种OPFRs使用范围较广,且容易通过多种途径接触大米样品。不同地区大米样品中OPFRs的种类存在一定差异,这可能与当地工厂中OPFRs的生产类型相关。检出化合物的各个特征碎片离子与自建标准谱库匹配较好,说明该谱库具有良好的适用性。

## 3 结论

本研究基于QuEChERS-GC-Q-TOF/MS建立了大米中21种OPFRs的筛查方法,通过对裂解机理进行探究,建立了OPFRs标准谱库,为实现靶向筛查大米样品中的OPFRs提供了主要依据。对样品前处理方法和仪器条件进行优化,通过设置特征碎片离子数量、精确质量窗口、保留时间偏差及碎片离子丰度偏差,确定了OPFRs的筛查流程,并对筛查方法进行了验证。利用解卷积软件对大米样品的全扫描数据进行处理和分析,提高了目标化合物与自建标准谱库的匹配分值。本方法实现了复杂样品中OPFRs的准确鉴定,对GC-Q-TOF/MS在复杂食品基质中的OPFRs分析及化合物鉴定提供了参考。
